# Supplementary material for: A Comprehensive Hydrothermal Co-Liquefaction of Diverse Biowastes for Energy-Dense Biocrude Production: Synergistic and Antagonistic Effects
Source: Int J Environ Res Public Health. 2022 Aug 23;19(17):10499. doi: 10.3390/ijerph191710499 (PMC9518380; doi:10.3390/ijerph191710499)
Supplement: Supplementary file 1 [file ijerph-19-10499-s001.zip › ijerph-1856515-supplementary.pdf]

**Supplementary**

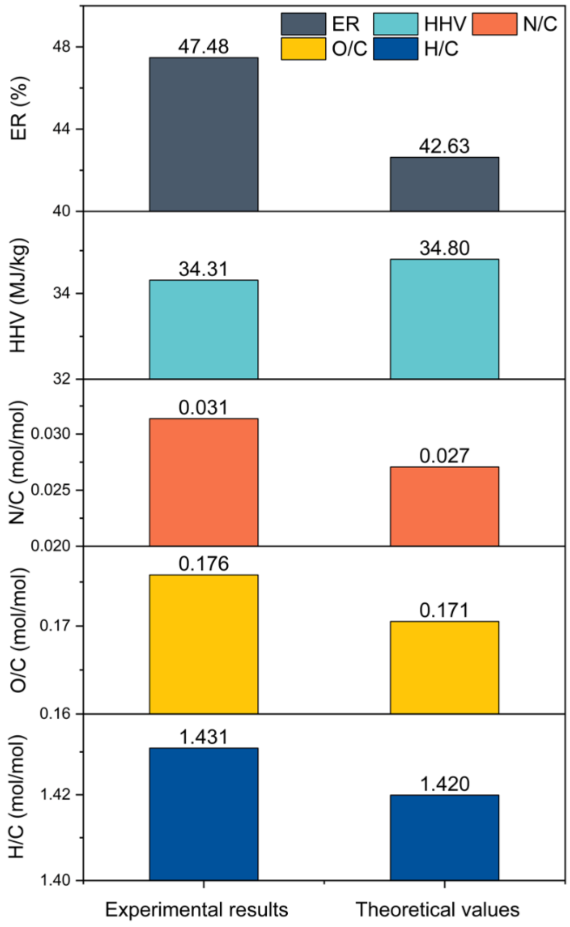


**Figure S1** Comparisons between experimental and theoretical results in terms of H/C ratio, O/C ratio and N/C ratio, HHV, and energy recovery of BC-SS/CM-30.

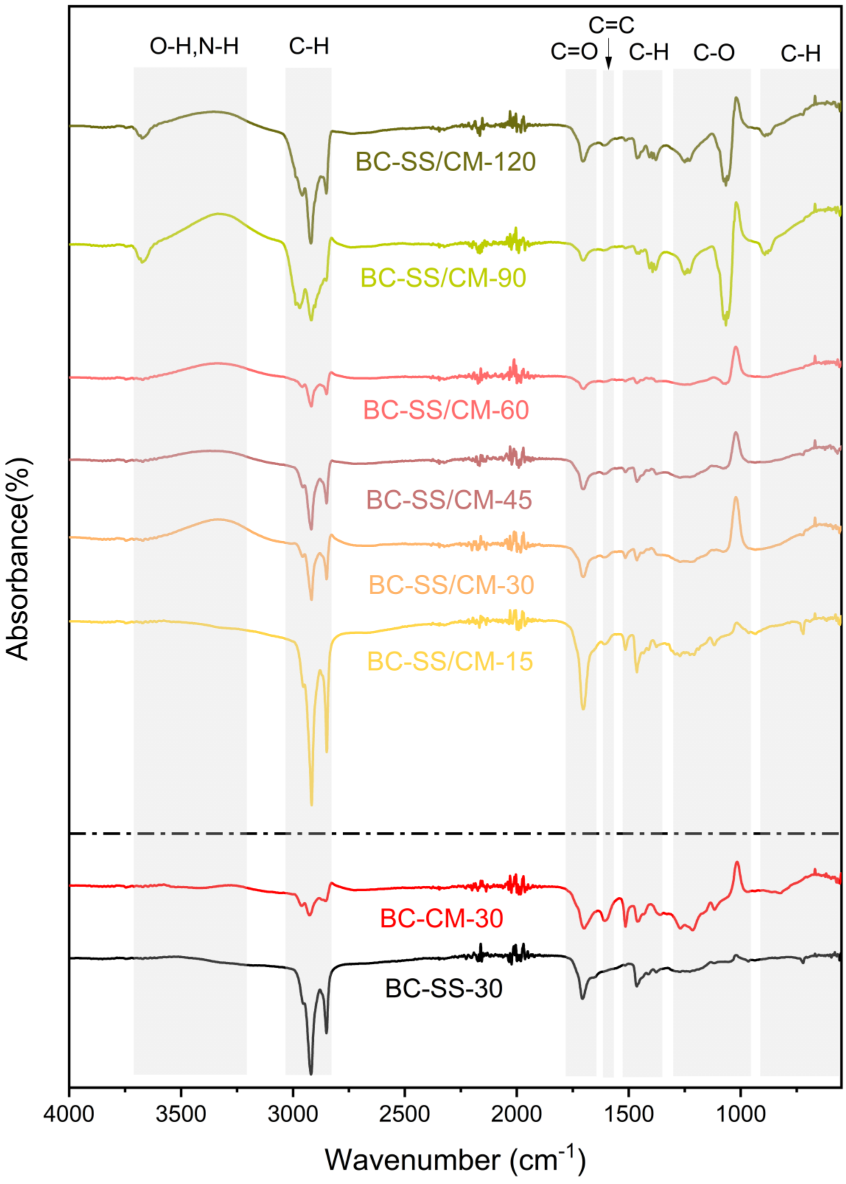


**Figure S2** FTIR spectra of biocrude oils from co-HTL of binary SS/CM with respect to residence time.

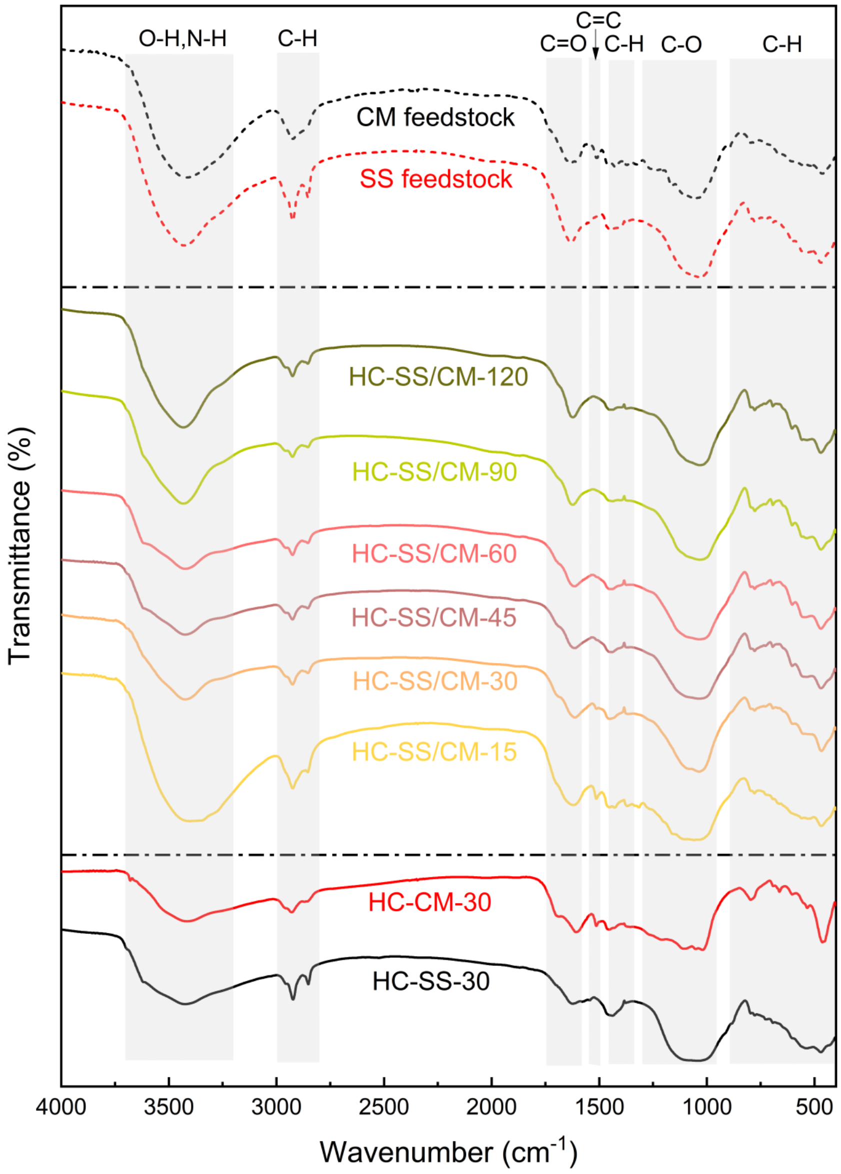


**Figure S3** FTIR spectra of biomass feedstocks and hydrochar samples from co-HTL binary SS/CM as a function of residence time.

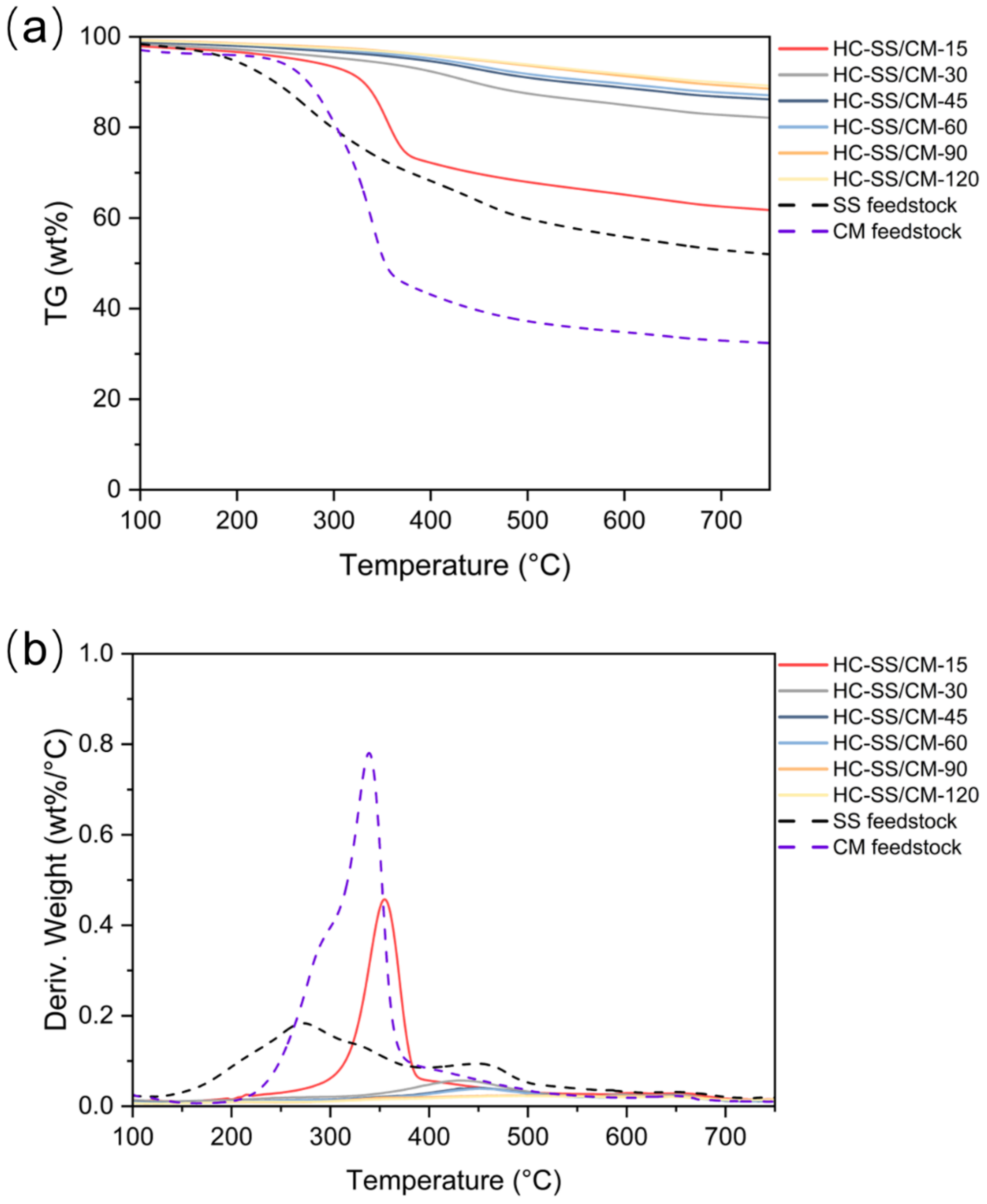


**Figure S4** TG-DTG curves of biomass feedstocks and hydrochar samples from co-HTL binary SS/CM as a function of residence time.

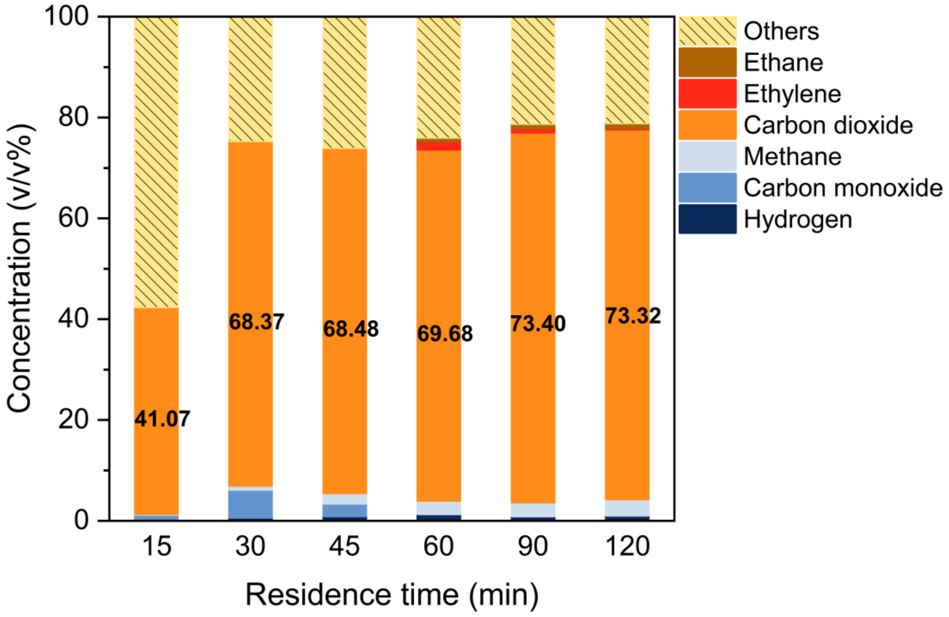


**Figure S5** The effect of residence time on gaseous compositions from co-HTL binary SS/CM.
